# Genetic and Environmental Interaction in Type 1 Diabetes: a Relationship Between Genetic Risk Alleles and Molecular Traits of Enterovirus Infection?

**DOI:** 10.1007/s11892-019-1192-8

**Published:** 2019-08-10

**Authors:** Marfa Blanter, Helena Sork, Soile Tuomela, Malin Flodström-Tullberg

**Affiliations:** 1 Center for Infectious Medicine, Department of Medicine Huddinge, Karolinska Institutet, Karolinska University Hospital, Stockholm, Sweden; 2 Laboratory of Molecular Immunology, Department of Microbiology and Immunology, Rega Institute for Medical Research, University of Leuven, Leuven, EU Belgium

**Keywords:** Autoimmune, Enterovirus, Environment, Genome-wide association studies, Immune system, Type 1 diabetes

## Abstract

**Purpose of Review:**

We provide an overview of the current knowledge regarding the natural history of human type 1 diabetes (T1D) and the documented associations between virus infections (in particular the enteroviruses) and disease development. We review studies that examine whether T1D-specific risk alleles in genes involved in the function of the immune system can alter susceptibility to virus infections or affect the magnitude of the host antiviral response. We also highlight where the major gaps in our knowledge exist and consider possible implications that new insights gained from the discussed gene-environment interaction studies may bring.

**Recent Findings:**

A commonality between several of the studied T1D risk variants studied is their role in modulating the host immune response to viral infection. Generally, little support exists indicating that the risk variants increase susceptibility to infection and moreover, they usually appear to predispose the immune system towards a hyper-reactive state, decrease the risk of infection, and/or favor the establishment of viral persistence.

**Summary:**

In conclusion, although the current number of studies is limited, this type of research can provide important insights into the mechanisms that are central to disease pathogenesis and further describe how genetic and environmental factors jointly influence the risk of T1D development. The latter may provide genetic markers that could be used for patient stratification and for the selection of method(s) for disease prevention.

## Introduction

Type 1 diabetes (T1D) is characterized by reduced insulin production due to a loss of the insulin-producing pancreatic β cells [1]. Disease onset typically takes place in childhood or young adulthood and in some countries the incidence is as high as 40–60 new cases per 100,000 children per year [2]. T1D is one of the most common chronic diseases in children and will, if left untreated, eventually lead to deadly coma [3]. Treatment consists of daily insulin administration and glucose monitoring. Most individuals with T1D have to cope with frequent hypo- and hyperglycemic episodes and have a high risk of developing various micro- and macrovascular complications associated with serious morbidity and a shortened life expectancy [4, 5]. While the etiology and pathogenesis of human T1D are not fully understood, both genetic and environmental factors seem to play an important role in regulating risk for disease development.

## The Natural History of Human T1D

Many observations point to the hypothesis that T1D is an immune-mediated disease involving the actions or regulatory failure of multiple compartments of the immune system. Prior to clinical disease manifestation, autoantibodies directed against β cell antigens are detectable in the blood of most individuals for a period of months to several years [1]. At disease onset, the pancreatic islets of most patients show hyperexpression of MHC class I [6•, 7] and many islets are infiltrated by immune cells [8, 9]. The immune infiltrate is mainly composed of CD8^+^ T cells, although CD4^+^ T cells, B cells, and cells of the innate immune system are also present [10–12]. Based on these observations, the most frequently presented hypothesis regarding how T1D arises states that β cells are destroyed by autoreactive CD8^+^ T cells and that the appearance of autoantibodies in the prediabetic period marks the initiation of the autoimmune disease process. However, data from a large body of recent work assessing the human pancreas near to or at disease onset have demonstrated that the β cells are still present in many patients (recently reviewed in [13•]), including those individuals who have lived with the disease for more than 50 years [14]. Moreover, the degree of insulitis seems to differ between patients and variation even exists in different lobes of a single patient’s pancreas [10, 15, 16]. Age-related differences in the immune cell composition of the islet infiltrates have also been identified [11, 12], and, intriguingly, the relative pancreas size is reportedly smaller both at disease onset and in autoantibody-positive subjects as well as first-degree relatives [17, 18]. These findings underscore the fact that there is still much to be learned about the natural history of human T1D.

## The Genetic Background of T1D

While it is as of yet unclear what the exact triggers of β cell damage and subsequent T1D development are, more is known on the factors regulating the risk of disease development. Family studies have revealed that T1D has a genetic component and it is estimated that around 50% of the risk for T1D is heritable. Epidemiological studies and research on monozygotic twins have suggested that environmental exposure and epigenetic modifications account for the rest [1, 19, 20]. As reviewed by Jerram and Leslie, it is often hypothesized that genetics determine the *predisposition* a person has for developing T1D, while the environment provides the *trigger* for disease onset [19].

Different methods including linkage analyses have been employed in the search for genetic determinants of T1D. Genome-wide association studies (GWAS), which have the power to identify single-nucleotide polymorphisms (SNPs) associated with disease, have revealed around 60 different loci associated with T1D. The contribution of most of these loci to T1D risk is relatively modest (odds ratio ≤ 2.0) [21–23], and for most of the loci, the risk-conferring SNP(s) remain undefined. Many of the GWAS candidate genes have important functions in the immune system and a majority of the genes are also expressed by pancreatic islet cells, including the β cells [24, 25].

The increasing amount of available SNP data has facilitated the development of algorithms to calculate so-called T1D genetic risk scores (T1D-GRS), which are based on the combination of several risk alleles and by this are able to, with increasing precision, predict who is at risk for developing T1D [25, 26]. Even better predictions are achieved when the T1D-GRS is combined with non-genetic information such as autoantibody positivity, body mass index and age [26, 27].

## Virus Infections as Triggers of β Cell Autoimmunity and T1D

Various environmental factors including dietary components, infections and gut microbial composition have been studied in relation to T1D development [28]. Among infectious agents, viral infections and in particular those from the enterovirus family have been identified as likely triggers of the disease [29, 30]. Several studies have reported a temporal association between respiratory infections and the appearance of autoantibodies [31, 32, 33•, 34•]. Moreover, a recent meta-analysis showed a significant association between infection with any type of virus during pregnancy and T1D development during childhood [35]. Yet another meta-analysis revealed a clear association between enterovirus infections and either islet autoimmunity or T1D [36]. Given that most viral infections will trigger the production of interferons (IFNs) [37], it is also of interest to mention that two independent studies have reported that the appearance of autoantibodies is preceded by a so-called IFN-related gene transcriptional signature in blood [38, 39]. Many additional observations have suggested a link between enterovirus infections and the appearance of islet autoantibodies or T1D (for extensive reviews, see [29, 30]), including studies showing that children newly diagnosed with T1D are more frequently seropositive for enteroviral RNA than healthy controls [40–42] and reports suggesting that enterovirus infection in the gut is more common in patients with T1D [43] or children with islet autoimmunity [44].

In addition to these observations, enterovirus proteins have been found in the human T1D pancreas at disease onset by immunohistochemistry and mass spectrometry [45, 46•, 47], and islets isolated from newly diagnosed T1D patients express IFN-stimulated genes [48•], with the latter being a potential indication of a viral infection [49•].

The epidemiological evidence and case studies are also supported by in vitro. experiments and proof-of-concept studies in animal models. For example, pancreatic β cells express several receptors used by enteroviruses to enter cells [50•, 51] and numerous enterovirus species have been shown to infect and have cytopathic effects in pancreatic β cells [51–53]. Also, Coxsackie B viruses (CVBs) can trigger disease in genetically permissive SOCS-1-tg animals [52] and accelerate the development of T1D in non-obese diabetic (NOD) mice [54, 55].

It should also be noted that some questions exist regarding the role of enterovirus infections in T1D. For example, countries with higher frequency of enterovirus infections seem to have a lower incidence of T1D. Moreover, certain developed countries report a rising incidence of T1D each year [2], despite no increases in the number of enteroviral infections with enterovirus [56]. In addition, some virus serotypes have been directly correlated with a reduced risk of developing T1D [57]. Besides these epidemiological observations, some critique has also been brought forward as to the usefulness of certain methodological tools used to detect viruses in the pancreata at disease onset and in long-standing T1D cases [58, 59]. Finally, not all studies have been able to confirm the presence of increased levels of enteroviruses in the human T1D gut [60].

Despite these issues, there is ample support for a role for virus infections in T1D [31, 32, 33•, 34•, 61], with evidence suggesting that infections with enterovirus species are capable of triggering β cell autoimmunity in predisposed individuals [36]. From the data that has been published on examining the link or missing link between enterovirus infections and T1D, a reasonable conclusion is perhaps that enterovirus infections contribute to some but not all cases of T1D.

## Enteroviruses

Enteroviruses are small single-stranded RNA viruses that are common all over the world. Although some species are known to cause serious diseases, most infections do not lead to severe symptoms in the general population. Examples of enteroviruses include Coxsackievirus A (CVA) and CVB, echoviruses, rhinoviruses, enterovirus 71 (EV71), and poliovirus [62, 63]. Enteroviruses typically spread via the fecal-oral route and from contaminated surfaces, although some viruses, including the rhinoviruses, spread via respiratory droplet aerosols and direct person-*to*-person contact. Shedding of the virus in stool can occur for weeks, even in asymptomatic individuals [64].

The key to survival following an enterovirus infection is an intact innate immune response, with the early production of type I IFNs being of critical importance (reviewed in [37]). IFNs are important pro-inflammatory cytokines that induce production of IFN-stimulated gene (ISG) products rendering the cell less permissive to infection. Studies in knockout animals have shown the importance of numerous ISGs with direct or indirect antiviral activity that prevent early virus replication and dissemination prior to the activation of the adaptive immune response. In addition, IFNs upregulate antigen presentation by human leucocyte antigen (HLA) class I and induce the production of chemokines that attract immune cells (reviewed in [37]). Neutralizing antibodies, which appear early in the disease process and persist for life, are essential for viral clearance and long-term immunity (e.g., [65]). CD4+ T cells are likely to be of importance for initiating an adequate antibody response, but the exact role(s) of CD8+ T cells remain to be defined [66, 67].

## From Association to Functional Insight in the Post-GWAS Era: Gene-Environment Interaction Studies, with Focus on Genes with Polymorphisms that Predispose to T1D

Gene-environment interaction studies aim to describe how genetic and environmental factors jointly influence the risk of developing a certain disease. They can also provide insights as to how certain SNPs affect the host response to infection both at the cellular and organism level. In relation to T1D and for the identification of individuals developing T1D with a possible enteroviral etiology, it may among other be useful to identify loci/SNPs that increase the risk of infection, enterovirus-induced inflammation, and/or enteroviral persistence. These studies may also reveal mechanisms that are central to disease pathogenesis and moreover provide measures to identify individuals who are likely to be at increased risk for developing virus-triggered or virus-accelerated T1D, allowing them to benefit from a preventive virus vaccine or antiviral treatment.

**Fig. 1:**
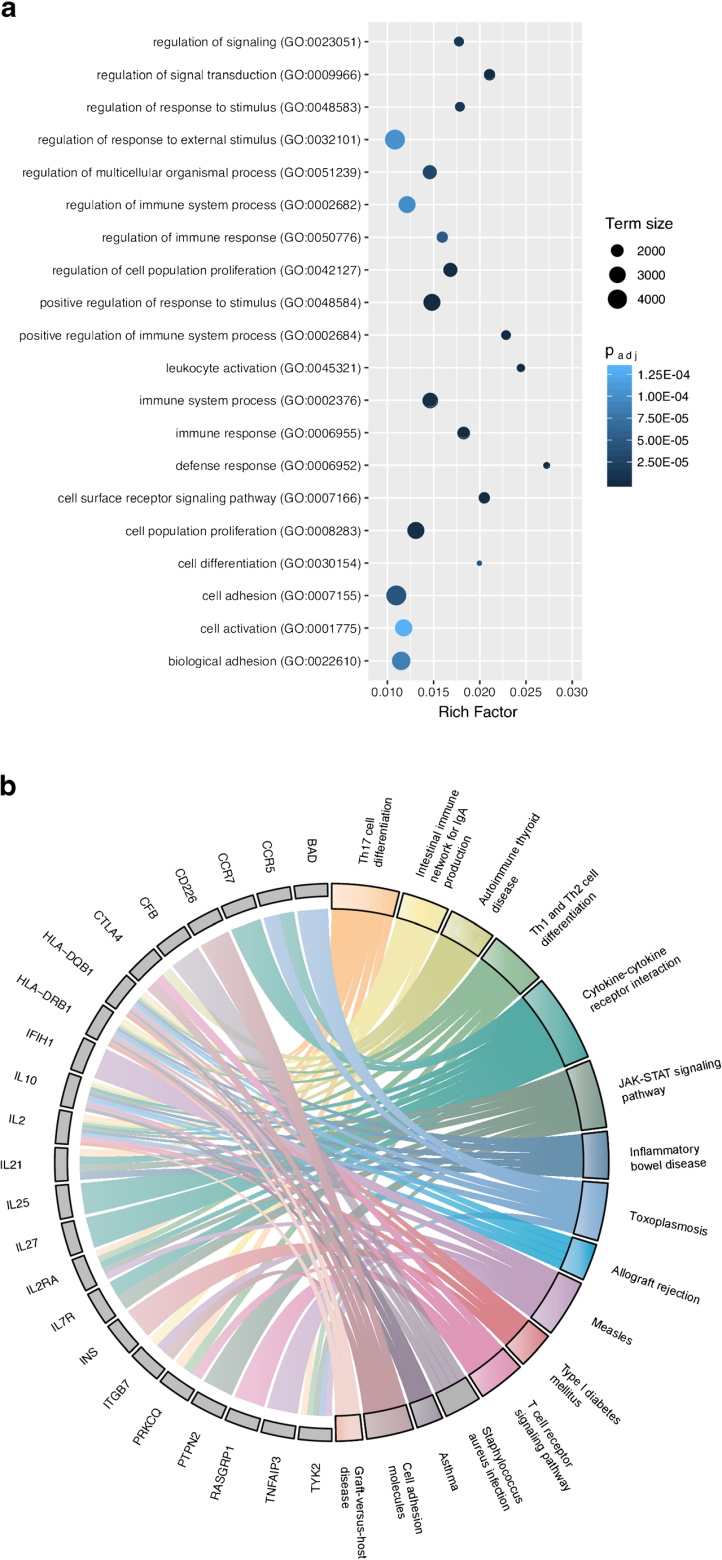
Gene Ontology (GO) and KEGG pathway analysis of T1D-related genes. **a** 118 genes obtained via the NHGRI-EBI Catalog and ImmunoBase were subjected to GO pathway overrepresentation analysis (“GO biological process”) using the program g:Profiler (Reimand et al., NAR, 2007) with default settings at Benjamini-Hochberg FDR of 0.05. The graph depicts the top 20 GO Slim categories (holding at least 1000 gene annotations) selected according to the lowest adjusted *p* value (p.adj). The genes categorized under GO:0002376 were used in the literature search to identify their involvement in viral infections and further subjected to KEGG pathway analysis (using g:Profiler) to gain a refined overview of their immune-related biological role. **b** The Circos plot depicts 23/46 of the genes (denoted with *gray boxes*) under GO:0002376 that had a KEGG pathway assigned (*colored boxes*). Graphs generated using the ggplot2 [143] and GOplot [144] packages in R, images modified using Inkscape 0.92 software

In this review, we describe several T1D risk genes that could be of interest in the context of virus-associated T1D development. To assist in identifying such genes, we compiled a disease-related gene list from GWAS data and an immunogenetic web resource through NHGRI-EBI Catalog [21] and ImmunoBase (https://www.immunobase.org/), respectively. Thereafter, we manually curated this list of genes to include immunologically relevant gene entries, which altogether resulted in a total of 118 genes. These genes could mainly be categorized under Gene Ontology (GO) pathways related to immune system processes, such as activation, proliferation, and signaling of immune cells, reflecting the autoimmune nature of the disease (Fig. 1a). We selected one of the major GO terms, GO:0002376 (“Immune system process”), which showed a highly significant enrichment in gene set enrichment analysis (had the second highest adjusted *p* value of all GO terms) and was among the terms that were comprised of the highest number of genes (46 genes in total). By this, we narrowed down our selection to these 46 genes and searched for published data implicating their involvement in viral infections. By retrieving published studies from the PubMed database (using the “virus OR enterovirus OR Coxsackievirus” search terms), we were able to identify that 36 of the 46 genes were related to viral infections, 15 of which had a connection to enterovirus-associated disease development and 13 being specifically associated with Coxsackievirus-related disease pathogenesis (Table 1). KEGG pathway analysis provided a glimpse of the biological pathways that several of the 36 genes are involved in (Fig. 1b)*.* As there seems to be a broader viral element in T1D disease initiation and progression than merely the enterovirus component [31, 32, 33•, 34•, 35], we aimed to provide an overview of a selected number of genes with published virus-associated disease phenotypes and discuss their potential involvement in T1D.

**Table 1:**
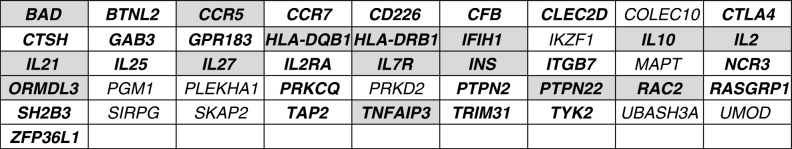
T1D-associated risk genes with a documented association with virus-associated traits

Overview of genes within the immune system process (GO:0002376) term and the presence of virus-related disease pathogenesis. PubMed literature search on the 46 genes within the GO:0002376 term revealed an importance of 36 genes in virus-associated pathogenesis (entries in bold). 15 of those revealed a connection to enterovirus-associated disease development (entries on gray background) with all the aforementioned (except for ORMDL3 and RAC2) being specifically indicated in Coxsackievirus disease pathogenesis.

### HLA class I and II

HLA class I and II molecules are involved in antigen presentation to CD8+ and CD4+ T cells, respectively. Little is known about the relationship between HLA class I risk alleles and enterovirus infection. Some enteroviruses have been shown to directly down regulate HLA class I on the surface of infected cells, thereby protecting them from recognition by CD8+ T cells [68–70]. Whether different HLA class I allelic variants exhibit a different level of sensitivity to this mechanism remains to be established. Individuals carrying the T1D low-risk allele B*5701 have been reported to have very low risk for progression of HIV-infection to AIDS (so-called elite controllers) [71]. Whether certain HLA class I alleles could provide similar protection from infections caused by viruses associated with T1D remains to be investigated.

The strongest risk for T1D has been attributed to HLA class II genes [20]. Multiple attempts have been made to connect HLA class II risk alleles to enterovirus infections [72–76]. Most groups have found no association between risk haplotypes and the presence of enteroviral RNA or neutralizing antibodies in the blood or stool, although one study has reported higher levels of anti-enterovirus antibodies in children bearing an HLA-DR risk allele, as compared to children that had a protective haplotype [77]. A review by Zhou and Jensen suggests that the mechanism behind the pathogenicity of HLA-DQ alleles is based on a higher affinity to a broad range of self-antigens [78]. A similar hypothesis was proposed by Marttila et al., who found that T cells derived from T1D patients with the high-risk allele DQB1*0302 responded to a broader range of viral epitopes compared to other patients [79]. Another risk allele, HLA-DR4, was found to be associated with a hyper-responsiveness of T cells to CVB4 antigens in vitro in cursive [80]. In contrast, Ellis et al. found no difference in binding of a common CVB4 epitope to HLA molecules derived from different alleles [81]. Hence, more research is needed to clarify the role of both HLA class I and II in enterovirus infections in humans.

### *IFIH1* (MDA5)

IFN induced with helicase C domain 1 (*IFIH1*) encodes the cytoplasmic pattern recognition receptor (PRR) melanoma differentiation-associated protein 5 (MDA5), which is a component of the innate immune system that recognizes double-stranded RNA (dsRNA). Upon detecting dsRNA, a hallmark of enterovirus replication, MDA5 initiates a signaling pathway that ultimately leads to the activation of the transcription factors NF-κB and IRF3, the transcription of and the subsequent production of type I IFNs [82]. Experimental studies have shown that MDA5 is an IFN-inducible gene (e.g., [83]) and an essential PRR for the detection of enterovirus infections [84, 85].

GWAS studies have identified common and rare gene variants of *IFIH1,* which regulate risk for T1D development [86, 87]. Consequently, many studies have investigated the functional aspect of *IFIH1* in T1D and also in enterovirus infections [82]. For example, two case-control studies found that *IFIH1* expression in peripheral blood cells was increased in T1D patients with risk variants of *IFIH1,* as compared to healthy controls and individuals without the risk allele [88]. Individuals heterozygous for the rs1990760 SNP, a common nonsynonymous SNP which has been strongly associated with altered risk for T1D development, were reported to be more often positive for enteroviral RNA in the blood, although the association was not very strong [89]. A similar study looking at enterovirus levels in the stool found no link with any of the common polymorphisms in *IFIH1* [90]. Pang et al. reported no association between the common SNP and the risk for enterovirus infection, but instead found a link with *severe* EV71 infection, indicating that the SNP may influence host immune response without affecting viral clearance [91]. Jermendy et al. reported that the risk SNP rs1990760 is associated with the seasonality of T1D disease onset, with summer being the season when the proportion of individuals carrying the homozygous risk genotype was highest over the calendar year [92••]. This discovery is interesting considering that summer is also the season when enterovirus infections peak [93].

Experimental studies have shown that mice which lack MDA5 show an increased susceptibility to enteroviral infection and higher mortality than wild-type animals [84, 85]. In contrast, heterozygous (MDA5+/-) NOD mice expressing lower levels of MDA5 than homozygous (MDA5+/+) NOD mice are protected from virus-induced T1D [94]. A mouse model with a knock-in mutation encoding IFIH1^T946^ showed enhanced production of type I IFNs at steady state and improved survival following lethal viral challenge but displayed greatly increased risk for autoimmune disease [95]. In addition, experimental studies have demonstrated that MDA5 encoded by variants of IFIH1 associated with protection from T1D development show loss of function [86, 87]. Collectively, these findings suggest that the presence of MDA5 is required for efficient antiviral defense, but that excess levels of MDA5 (and increased production of IFNs) may contribute to inflammation and autoimmunity.

### PTPN22

Protein tyrosine phosphatase non-receptor type 22 (*PTPN22*) contains risk polymorphisms that almost double the risk for T1D. PTPN22, also known as Lyp, is a tyrosine phosphatase expressed in hematopoietic cell lineages. PTPN22 plays an inhibitory role in the activation of the immune system by inhibiting T cell receptor signaling and preventing the expansion of effector T cells [96]. In addition, PTPN22 has been reported to positively regulate TLR-triggered IFN production in myeloid cells [97], suggesting that it contributes both to innate and adaptive immune functions.

The most well-studied polymorphism in *PTPN22* is rs2476601, which causes a substitution of an arginine by a tryptophan at position 620 (R620W). This polymorphism has been associated with T1D in most population groups, as well as with other autoimmune diseases. The R620W polymorphism most likely causes a functional change in the PTPN22 protein. Though there is currently no consensus within the field which mechanism underlies the altered risk for autoimmunity in individuals carrying this polymorphism, suggestions have been made that it may be context and/or cell type dependent [96].

Interestingly, a few studies report the involvement of *PTPN22* in infections with viruses other than enteroviruses. Wang et al. showed that the rs2476601 SNP is associated with lower IFN production by macrophages in response to TLR ligand stimulation and suggested that this may result in a weakened antiviral response to infections [97]. Crabtree et al. found that the T1D rs2476601 risk allele is associated with reduced CD4+ T cell response and antibody affinity maturation to influenza vaccination [98]. Maine et al. identified PTPN22 as the key promoter of chronic infection with lymphocytic choriomeningitis virus (LCMV) by suppressing T cell activation [99]. In contrast, Montes-Cano et al. did not observe an effect of the rs2476601 SNP on the outcome of chronic hepatitis C infection [100]. Thus, it could be that by suppressing the function of effector T cells, PTPN22 diminishes response to certain virus types, allowing the establishment of a persistent infection. However, the suppressive effect of the rs2476601 on the immune system as proposed by Crabtree et al. [98] is in direct contradiction with the suggested mechanism in T1D, i.e., reduced inhibition of effector T cells or increased suppression of regulatory T cells (Tregs) [96].

The MIDIA study has looked at the relationship between *PTPN22* and enterovirus infection [101]. The study had an epidemiological setup, in which a large group of Norwegian children were screened for SNPs in T1D risk alleles. The genotypes were then correlated to the presence of enterovirus in stool samples. Although the rs2476601 SNP had one of the highest correlations with enterovirus positivity, this association was not strong enough to reach statistical power. Hence, more research is needed to gain better insights into the functional consequences of the rs2476601 SNP and its involvement in T1D and enterovirus infection.

### CTLA-4

Cytotoxic T lymphocyte-associated protein 4 (*CTLA-4*) encodes the CTLA-4 protein, also known as CD152. CTLA-4 is a molecule that is normally expressed in low levels on CD4+ and CD8+ T cells. CTLA-4 is structurally related to CD28, a stimulatory molecule that contributes to effector T cell activation by binding the B7 ligand expressed on antigen-presenting cells (APCs). This binding is necessary for the activation to occur. CTLA-4 competes with CD28 for B7 molecules, and high expression of CTLA-4 leads to inhibition of T cell activation [102].

A few studies have been published on the connection between *CTLA-4* and enterovirus infections. Thus, a study by Yang et al. found an association between a T1D risk associated SNP in *CTLA-4* (rs231775) and the likelihood of developing severe meningoencephalitis after infection with EV71 [103]. A similar finding was done by Han et al., who showed that the administration of CTLA-4 fusion protein attenuated Coxsackievirus B3-induced myocarditis in mice [104]. Since viral myocarditis is for a large part caused by excessive inflammation rather than virus-induced cytopathology [105], both studies are suggestive that CTLA-4 mostly affects the immune response to the pathogen rather than the viral entry or replication. These observations together with the demonstration that T1D-related *CTLA4* polymorphisms are linked to increased IFN-γ production by peripheral blood mononuclear cells in response to a broad range of different antigens, including CVB4 [106], suggest that autoimmunity associated SNPs in *CTLA4* are coupled with impaired immunoregulation, which may elevate the risk for immunopathology during enterovirus infection.

### IL10

The interleukin-10 gene (*IL10*) encodes the cytokine IL-10, which is produced predominantly by Tregs and has an anti-inflammatory effect [107]. SNPs in the *IL10* gene region have been identified in GWAS studies as risk factors for T1D [23].

It has been reported that infections with CVB3 and CVB4 are associated with increased IL-10 production [108–111]. A similar association has also been described for EV71 [112–114]. The relationship between SNPs in the *IL10* gene region and infections with enterovirus has also been examined in several studies. The rs1800896 SNP—a nucleotide substitution upstream of the *IL10* gene—has been associated with various inflammatory diseases, including autoimmune liver disease [115] and systemic lupus erythematosus [116], and is strongly correlated with susceptibility to EV71 infections in Chinese children [117]. Another SNP in the promoter region of *IL10*, rs1800872, was found to increase the risk of EV71-caused hand, foot, and mouth disease [118].

Several studies suggest that IL-10 may promote viral persistence [107]. For instance, Yeung et al. found that children positive for enteroviral RNA usually had elevated serum levels of IL-10. However, this did not correlate with their T1D status [119]. Furthermore, an experimental study showed that IL-10 production was induced in mice upon infection with CVB and appeared to facilitate the development of chronic pancreatitis, as IL-10 knockout animals resolved the acute pancreatitis [120].

### TNFAIP3

TNF-alpha-induced protein 3 (TNFAIP3), also known as A20, is a cytoplasmic protein which mainly exerts anti-inflammatory function by inhibiting NF-κB activation [121]. SNPs in *TNFAIP3* have been associated with multiple autoimmune diseases, including T1D [23]. Even prior to GWAS, *TNFAIP3* was identified as a T1D risk allele, and in vitro studies discovered that A20 expression can be induced in β cells to protect them from cytokine-induced apoptosis. A subsequent animal study reported that genetic therapy with A20 could protect against T1D in a streptozotocin-induced T1D mouse model [121]. In 2016, Fukaya et al. reported that the noncoding rs2327832 polymorphism in *TNFAIP3* is associated with reduced residual β cell function and impaired glycemic control in children with T1D [122]. This same polymorphism was later confirmed to have an association with a higher susceptibility to T1D in a large cohort [123].

Very little research has examined the role of A20 in enterovirus infections. It has been reported that A20 ameliorates CVB3-induced myocarditis by inhibiting NF-κB signaling [124]. Interestingly, Doukas et al. reported that *TNFAIP3* is one of the few genes that escape transcriptional shutoff by the poliovirus. In addition, the authors report that depletion of A20 leads to increased virus replication [125]. In contrast, some other virus types (e.g., influenza A), profit from high A20 expression, since they can inhibit the expression of type I IFNs [126, 127].

### Interleukin-21 and IL-2

The interleukin-21 (*IL21*) gene encodes IL-21, a cytokine that is mainly produced by T cells and natural killer T cells [128]. It acts on both lymphoid and myeloid cells, and has been associated with positive and negative immune regulation depending on the context. The *IL21* locus is shared with the interleukin-2 (*IL2*) gene, encoding a cytokine that is involved in the activation of effector T cells and in the generation, homeostasis, and function of Tregs [129]. Both genes have been shown to play a role in autoimmunity; IL-21 by promoting T follicular helper- (Tfh) and Th17 cell differentiation, and by inhibiting Treg generation, and IL-2 via altered Treg function and homeostasis [130–132].

As shown by Yeung et al., children infected with enteroviruses generally have lower serum concentration of IL-21 than those that are not infected, although this did not correlate with their T1D autoantibody status (i.e., prediabetic or healthy) [119]. Another study investigated IL-21 signaling in the context of CVB3-induced myocarditis and concluded that IL-21 mediate excessive activation of CD8+ T cells, thereby contributing to inflammation [133]. Importantly, the increase in effector T cells does not lead to a reduced virus titer, which is rather indicative of a hyper-reactive immune system without increased functionality. Consistent with this finding, mice deficient in the IL-21 receptor display lower inflammation of the myocardium and fewer autoreactive B cells, lowering tissue damage in virus-induced myocarditis [134].

*IL2*, the second gene in the IL-21 gene cluster, is predominantly expressed in activated T cells. IL-2 binds to its receptor, IL-2R, and stimulates proliferation of both effector and regulatory T cells [129]. GWAS studies have also identified T1D associated SNPs in the *IL-2RA* gene and linked them to altered IL-2R signaling in Tregs [131]. Serum IL-2 levels do not differ between T1D and healthy controls [135], but it has been reported that they are elevated in severe EV71-induced hand, foot, and mouth disease [136]. In viral myocarditis, IL-2 has been shown to play a dual role, contributing to improved viral clearance during the acute phase and prolonging inflammation during the chronic phase [137].

## Implications of Gene-Environment Interaction Studies

GWAS have already revealed many important aspects of T1D etiology. With the emergence of genome-editing techniques, we have entered the post-genomic era with the possibility to introduce specific changes into cell lines and primary cells, study the independent and combined effect(s) of SNPs on biological functions, and also identify causal SNPs. Such efforts may also provide insight into mechanisms that are central to virus-triggered T1D and facilitate the search for therapeutic targets.

Insights gained from gene-environment interaction studies coupled with further genetic studies on phenotypically defined subgroups of T1D patients may also assist in refining the current T1D-GRS, and perhaps even more importantly, open the option of stratification of individuals based on disease endotypes. For this to become a reality, further insights into the genetic, as well as non-genetic, determinants of disease heterogeneity may be needed. Moreover, we may need to consider that genes and environmental factors of importance for the triggering events (i.e., induction of islet autoimmunity) may not be the same as those involved in precipitating disease in already autoantibody-positive individuals (i.e. clinical disease development). For the identification of an endotype with an enteroviral etiology, we also need to learn more about when and how the virus contributes to the disease. While the occurrence of virus in the pancreas at disease onset has led to the hypothesis that the virus causes a persistent infection in the islets, it is as of yet unclear whether unresolved infections, acute infections, or both are accountable for the reported link between enterovirus infection and T1D.

Another aspect to consider for the development of GRS is population diversity. Currently, most of the GWAS studies have been performed in the Caucasian populations living in Europe or North America. We are just in the beginning of understating how translatable the GRS are to other regions in the world, or even to other ethnicities living in Europe or North America [138]. Beyond this, and to further improve the precision of the GRS, genome-wide association interaction studies (GWAIS) will most likely be required to unravel functional relationships between genes (epistasis) [139].

Among the non-genetic factors to be taken into account when calculating risk for T1D development, age may be of importance as twin studies for example have indicated that the heritable risk component declines with age [140]. For predicting T1D with an enteroviral etiology, gender may also play a small, yet non-negligible role [41].

Due to these additional layers of complexity, SNP genotyping and calculation of GRS might not prove to be a feasible strategy for predicting the absolute risk of developing T1D, although it might be useful for risk assessment and patient stratification in case-control studies and clinical trials. Hence, these measures could also be used to stratify prediabetic individuals who would benefit from receiving an enterovirus vaccine, or dividing recent onset T1D patients into subgroups that would be more or less likely to respond favorably to antiviral therapy. Enterovirus infections could be treated with antivirals and many efforts are being made to develop broadly acting antivirals (reviewed in [62, 129]). While a vaccine against enteroviruses suspected to play a role in triggering T1D is not yet available, prototype vaccines against CVBs have shown excellent safety, immunogenicity, and efficacy in preventing infection and enterovirus-associated disease [54, 141, 142•].

## Conclusions

This review summarizes current knowledge surrounding the relationship between enterovirus infections and T1D, with a special focus on T1D genetic risk alleles in genes related to the host immune system and their possible role in altering susceptibility to virus infection or the magnitude of the host antiviral immune response. It is evident that these types of study have so far been limited in number and few have examined the direct interactions between T1D-associated genes/SNPs and the outcome of enterovirus infections. The studies cited do not support that SNPs in the genes which have been reviewed are increasing susceptibility to infection, more often they seem to either decrease the risk for infection or favor the establishment of viral persistence. This fits well with the chronic nature of islet autoimmunity. The studies also point to the fact that several T1D-associated genes modulate the immune response to virus infection. Indeed, several risk variants appear to predispose the immune system towards hyper-reactivity, which may manifest itself upon encounter with an exogenous pathogen, such as an enterovirus, and thereby trigger autoimmunity.

Further research in this area will provide additional insights into the etiology and pathogenesis of T1D. This may not only assist in defining therapeutic targets, but also provide measures to identify individuals at risk—such studies can be translated into clinically useful advances in the personalization of T1D prevention by providing genetic markers useful for patient stratification and the selection of method(s) for disease prevention.
